# In‐Field Ecological Momentary Assessment From Wearable Motion Sensors and Self‐Report in a Chronic Low Back Pain Cohort

**DOI:** 10.1002/jsp2.70156

**Published:** 2026-02-01

**Authors:** Kevin M. Bell, Zakiy Alfikri, William Anderst, William W. Clark, Harold A. Cook, Jessa Darwin, Brad E. Dicianno, Carol M. Greco, Jordan Hoydick, John M. Jakicic, Gina P. McKernan, Bambang Parmanto, Charity G. Patterson, Sara R. Piva, Rachel E. Roos, Michael J. Schneider, Meenakshi Sundaram, Nam V. Vo, Leming Zhou, Gwendolyn A. Sowa

**Affiliations:** ^1^ Department of Bioengineering University of Pittsburgh Swanson School of Engineering Pittsburgh Pennsylvania USA; ^2^ Department of Orthopaedic Surgery University of Pittsburgh School of Medicine Pittsburgh Pennsylvania USA; ^3^ Mechanical Engineering and Materials Science Department University of Pittsburgh Pittsburgh Pennsylvania USA; ^4^ Department of Physical Medicine and Rehabilitation University of Pittsburgh School of Medicine Pittsburgh Pennsylvania USA; ^5^ Department of Psychiatry University of Pittsburgh School of Medicine Pittsburgh Pennsylvania USA; ^6^ Department of Physical Therapy University of Pittsburgh School of Health and Rehabilitation Science Pittsburgh Pennsylvania USA; ^7^ Department of Internal Medicine University of Kansas Medical Center Kansas City Kansas USA; ^8^ Department of Biomedical Informatics University of Pittsburgh School of Medicine Pittsburgh Pennsylvania USA; ^9^ Clinical and Translational Science Institute, University of Pittsburgh Pittsburgh Pennsylvania USA; ^10^ Doctor of Chiropractic Program University of Pittsburgh School of Health and Rehabilitation Science Pittsburgh Pennsylvania USA; ^11^ Ferguson Laboratory for Orthopaedic and Spine Research, Bethel Musculoskeletal Research Center, Department of Orthopaedic Surgery University of Pittsburgh School of Medicine Pittsburgh Pennsylvania USA; ^12^ Department of Pathology University of Pittsburgh School of Medicine Pittsburgh Pennsylvania USA; ^13^ McGowan Institute, University of Pittsburgh Pittsburgh Pennsylvania USA; ^14^ Department of Health Information Management University of Pittsburgh School of Health and Rehabilitation Sciences Pittsburgh Pennsylvania USA; ^15^ Intelligent Systems Program University of Pittsburgh School of Computing and Information Pittsburgh Pennsylvania USA

**Keywords:** chronic low back pain, ecological momentary assessment, physical activity, sleep, wearable motion sensors

## Abstract

**Background:**

Chronic low back pain (cLBP) is a prevalent and debilitating condition. Gaining insight into the daily experiences of those with cLBP is crucial for developing effective management. Pain and activity are typically assessed at a single time point and often rely on retrospective self‐reports, which can be prone to recall bias and may not reflect the day‐to‐day variability of these experiences. As a part of the University of Pittsburgh LB^3^P Mechanistic Research Center, this study used ecological momentary assessment (EMA) and wearable devices to collect real‐time data in a large cohort of adults with cLBP. The primary aims were to collect and characterize pain and activity profiles of individuals with cLBP.

**Methods:**

This study enrolled 1007 adults with cLBP who met the National Institutes of Health defined criteria. Over 7 days, participants were assessed in their own environment. EMA was gathered in real‐time via a custom mobile app, prompting participants three times daily to provide their perceptions of current pain intensity (0–10), pain interference (0–10), and activity level (very light to vigorous). Time of falling asleep and waking was also reported. Participants wore ActiGraph GT9X devices on their wrist and waist. A custom back sensor was also adhered to the skin over the lumbar (L5) segment. Activity counts, wear time, and step counts were calculated, utilizing algorithms provided by ActiGraph. Sensor data were filtered to include at least 4 days of 10 or more hours each. Activity counts were categorized into sedentary, light, and moderate‐to‐very‐vigorous based on Freedson Adult cutpoints.

**Results:**

Out of 1007 participants, 989 submitted EMA data (58.8 ± 16.5 years old; 40% male and 60% female; mean pain intensity at enrollment of 5.4 (SD 2.1) and a median of 5 (interquartile range [IQR] 3) on a 0–10 scale; mean PROMIS Pain Interference *T*‐score at enrollment of 60.5 (SD 7.5) and a median of 61.2 (IQR 9.6)). The median reported pain intensity level from the EMA was 1 (IQR = 3), while pain interference was 3 (IQR = 3). More than half of the participants reported a median pain intensity of either 0 or 1 (54.0%) and a median pain interference between 0 and 3 (57.4%). Most participants self‐reported their activity levels as moderate (36%) or light (33%). Based on pain ratings during each day, most participants had their pain intensity (30%) and pain interference (40%) peaking in the evening. ActiGraph data from 884 wrist‐worn and 785 waist‐worn devices were analyzed. Wrist data showed a median of 1 765 325 (IQR 796 995) activity counts/day and 9575 (IQR 4228) steps/day. Waist data showed 358 390 (IQR 223 758) activity counts/day and 4114 (IQR 3146) steps/day. The percentage of daily sedentary activity was 47.3% for wrist and 72.8% for waist. The back sensor data from 586 participants showed a median of 340 345 (IQR = 223 399) activity counts/day and a median of 3695 (IQR = 2743) steps/day. The percentage of time spent in daily sedentary activity was 82.6%. Both ActiGraph devices and the back sensor indicated that the majority of the time was spent in sedentary activity level, which is lower than the activity level reported in the EMA.

**Conclusions:**

Despite having cLBP with self‐reported moderate pain levels, participants generally reported periods of relatively low levels of pain intensity and interference in their EMA. In addition, their EMA‐reported activity levels differed from the sensor data. Participants self‐reported higher levels of activity compared to the activity levels calculated by the wearable sensors. This suggests that participants overestimated their activity levels on EMA, or that the activity level cut‐points may need to be re‐evaluated for the cLBP population. Additionally, sensors placed on different body locations showed varying activity and step counts. The activity counts calculated from the waist ActiGraph and the back sensor from this cohort were lower than the average activity counts in the US adult population. Further research is needed to better quantify these differences for people with cLBP to develop a more comprehensive understanding of the pain experience.

## Graphical abstract

In a large cohort of adults with chronic low back pain, real‐time ecological momentary assessment and multisite wearable sensors were used to characterize daily pain and physical activity in participants' natural environments. While participants frequently reported low pain intensity and moderate activity levels, objective sensor data from the wrist, waist, and lumbar spine revealed predominantly sedentary behavior and lower activity levels, highlighting discrepancies between perceived and measured activity.

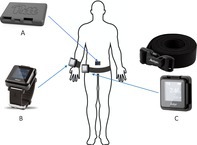

## 1 Introduction

Individuals with chronic low back pain (cLBP) often experience significantly reduced activity levels [1] and high levels of sedentary behavior [2]. They struggle to engage fully in both work and leisure activities [3, 4]. cLBP has also been shown to impact other lifestyle factors such as sleep duration and sleep quality [5]. There are a variety of causes for the reduction in physical activity levels including physical barriers such as pain intensity and comorbidities [6], fear of reinjury [7], which can lead to a vicious cycle of disuse and deconditioning leading to worsening function [8]. Therefore, for people with cLBP, monitoring and encouraging physical activity is prioritized in a biopsychosocial treatment approach [9].

Understanding the daily experiences of individuals with cLBP is crucial for characterizing the impact of cLBP and developing effective interventions. Ecological momentary assessment (EMA) is a method of collecting data in the real world, in real time, and often uses mobile technology [10, 11]. EMA data sources can include both self‐reported questionnaires and objective data from wearable sensor technologies. EMA is particularly effective for longitudinal assessment [12]. It records data that varies over time and space as events happen, thereby improving the granularity of changes and trends over time and minimizing recall biases. A variety of EMA data sources can be integrated together (i.e., self‐report, wearable sensors) to provide a more comprehensive picture of functional activities of an individual or population of individuals [10].

Wearable technologies and tools for tracking physical activity metrics have seen a notable rise in focus over recent years. For cLBP, these devices are typically worn on the wrist, waist, or lower back and the most common wear duration is 7 days [11]. Most wearable technologies incorporate accelerometers or inertial measurement units (IMUs), which measure and report acceleration, along with metrics like angular velocity, magnetic field, and orientation estimates. High variability in device models, wear duration, and wear locations makes comparison across studies challenging, potentially hindering research and clinical advancements. Hence, integrating multiple EMA and wearable technologies from a variety of sources in a large cohort of individuals with cLBP may provide new insights into the response to treatment or phenotyping in large populations [13]. The objective of this study was to utilize complementary self‐reported EMA and wearables to characterize pain characteristics, physical activity, sedentary behavior, and sleep patterns from individuals with cLBP in the field over a 7‐day period, presenting results both for the entire cohort and stratified by age (≥ 60 years old, < 60 years old) and sex at birth (male, female).

## 2 Materials and Methods

This observational study reports data from the University of Pittsburgh's Low Back Pain: Biological, Biomechanical, Behavioral Phenotypes (LB^3^P) Mechanistic Research Center (MRC). The LB^3^P MRC is a member of the National Institutes of Health's (NIH) Back Pain Consortium (BACPAC) Research Program—which is part of the Helping to End Addiction Long‐term (HEAL) Initiative. The overall objective of LB^3^P is to perform in‐depth phenotyping of patients with cLBP using a multimodal assessment approach that can inform improved treatments. This study received approval from the University of Pittsburgh Institutional Review Board. A total of 1007 adults with cLBP were enrolled. Participants were recruited through various channels, including clinical care settings, research registries, and public announcements.

### 2.1 Participants

Participants were included if they were adults, English speakers, and had cLBP as defined by the NIH Task Force [14]—pain located between the inferior border of the ribcage and the gluteal fold for at least 3 months, with pain occurring on at least half the days in the past 6 months. Participants were excluded if they: (1) were not identified in the University of Pittsburgh Medical Center (UPMC) Electronic Health Record system, (2) were participating in a masked intervention study for LBP, or (3) had a medical condition that would place the participant at increased risk or preclude them from complying with study procedures. An in‐depth summary of the participants' demographics and biomedical characteristics is provided elsewhere [15].

### 2.2 Materials and Procedures

The study used data collected over a 7‐day period following participants' enrollment visit. Descriptions of the data collection and processing of the biomechanical data were previously published by the BACPAC Biomechanic Working Group [16].

**FIGURE 1:**
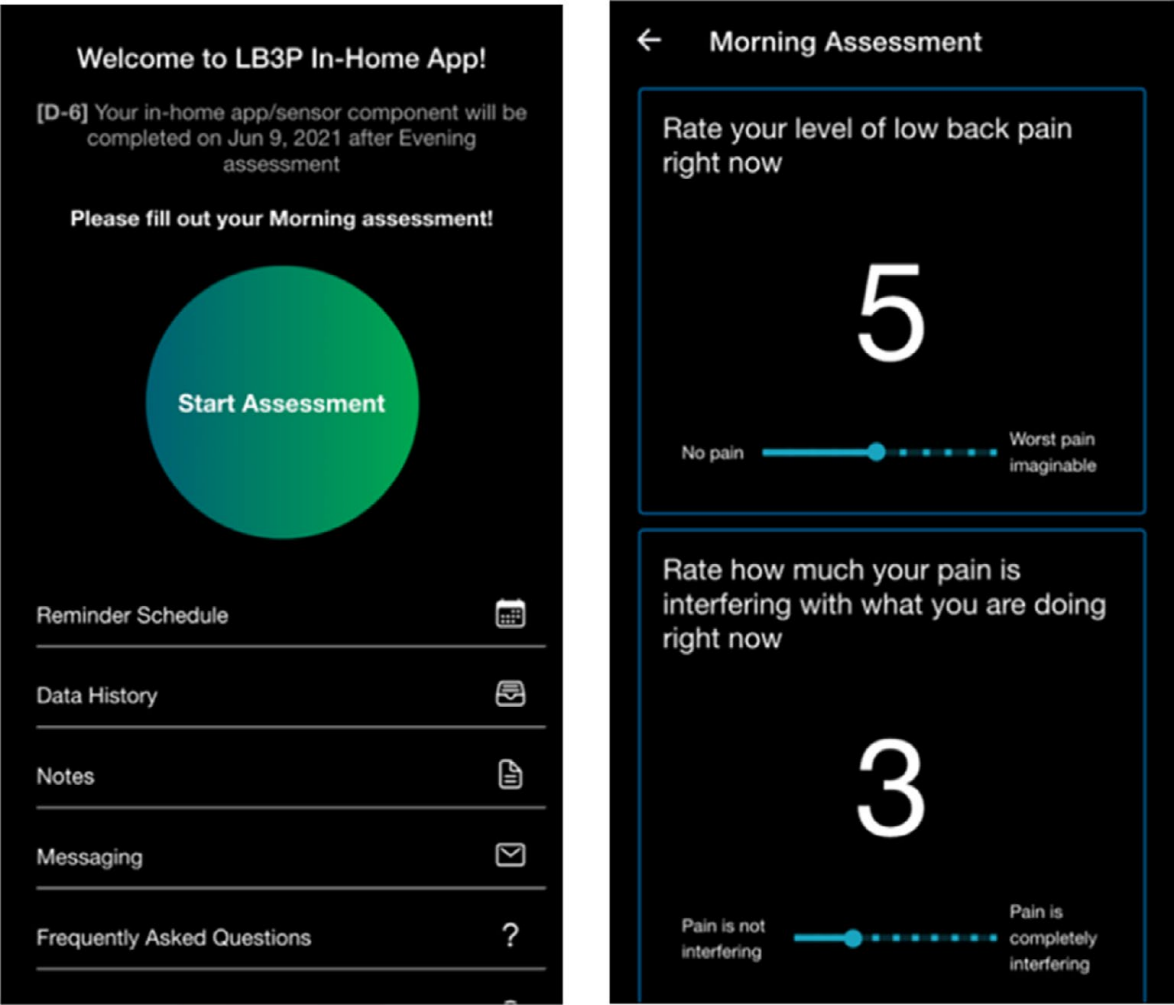
Screenshots of the custom app used to capture EMA.

Self‐reported EMA on pain and physical activity was gathered in real‐time in the participants' natural environment. The data collected through the self‐reported EMA included pain intensity levels, pain interference levels, activity levels, sleeping time, and waking up time information (Table 1). This data collection was facilitated by a custom mobile app (LB3P Toolbox), specifically developed for this study (Figure 1), which participants installed on their smartphones, whether Android or iPhone. For those without a smartphone, an Android phone (Samsung Galaxy A21) with network service was provided. The app prompted participants to complete the EMA three times daily, according to the specific schedule outlined in Table 1.

**TABLE 1:** Specific questions asked in the EMA.

Question	Answer options
Morning assessment (12 a.m.—12 p.m.; default notification set at 8 a.m.)	
Rate your level of low back pain right now	0 (No pain)–10 (Worst pain imaginable)
Rate how much your pain is interfering with what you are doing right now	0 (Pain is not interfering)–10 (Pain is completely interfering)
What time did you fall asleep?	Time
What time did you wake up?	Time
Afternoon assessment (12 p.m.–6 p.m.; default notification set at 1 p.m.)	
Rate your level of low back pain right now	0 (No pain)–10 (Worst pain imaginable)
Rate how much your pain is interfering with what you are doing right now	0 (Pain is not interfering)–10 (Pain is completely interfering)
What activities did you do this morning?	– Sports/Exercise – Hobbies – Work, school, or volunteer – Home activities
How much effort did your activities require?	– Very light – Light – Moderate – Moderate to vigorous – Vigorous
Evening assessment (6 p.m.–12 a.m.; default notification set at 7 p.m.)	
Rate your level of low back pain right now	0 (No pain)–10 (Worst pain imaginable)
Rate how much your pain is interfering with what you are doing right now	0 (Pain is not interfering)–10 (Pain is completely interfering)
What activities did you do this afternoon?	– Sports/Exercise – Hobbies – Work, school, or volunteer – Home activities
How much effort did your activities require?	– Very light – Light – Moderate – Moderate to vigorous – Vigorous
Was this a typical day for you?	Yes or No

**FIGURE 2:**
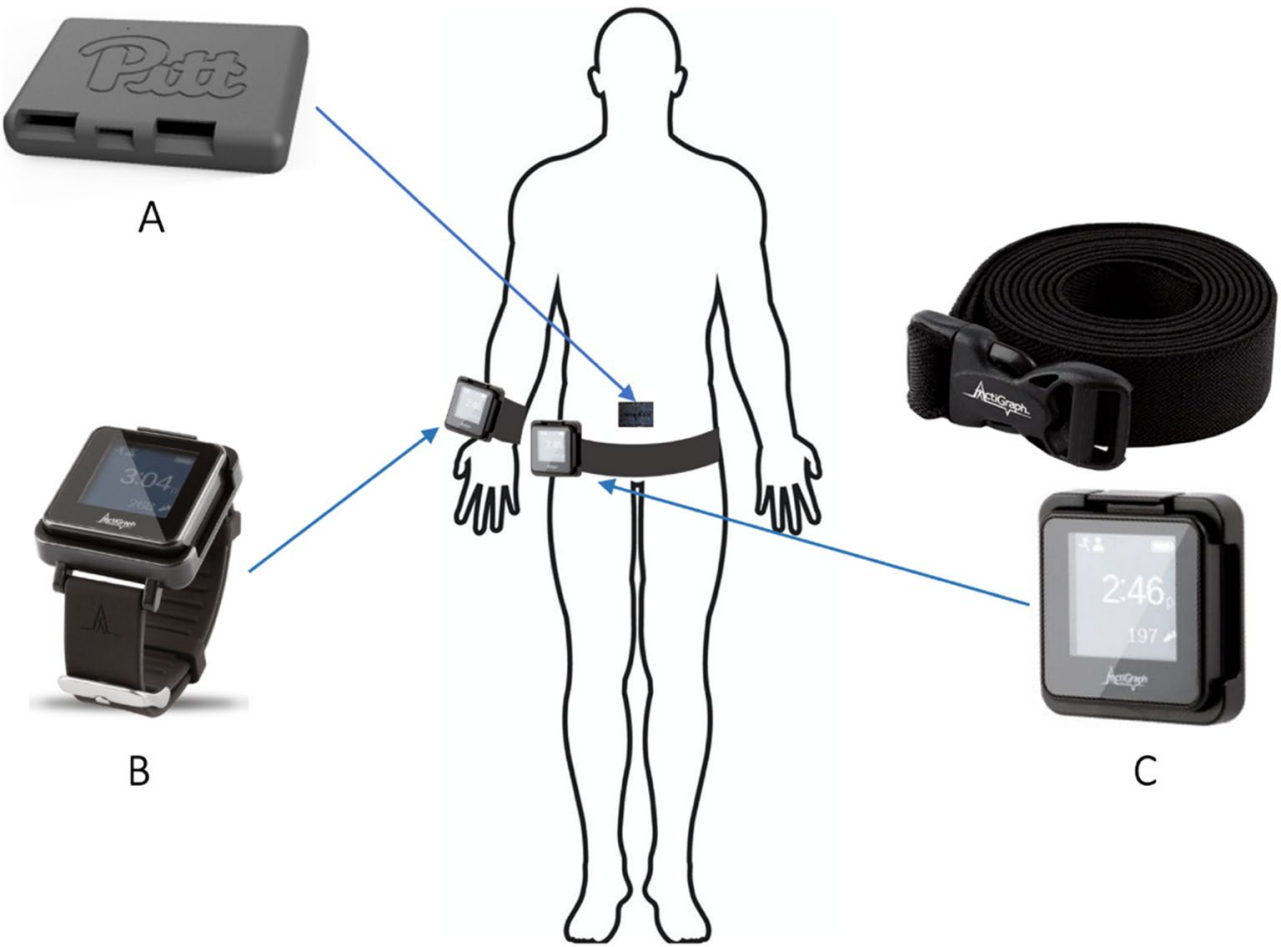
The sensors used for the at‐home assessment and their placement on the body (posterior view): (A) Custom back sensor placed at the L5 lumbar segment; (B) ActiGraph GT9X device with a watch band worn on wrist (source: https://theactigraph.com/); and (C) ActiGraph GT9X device with a belt worn on waist (source: https://theactigraph.com/).

A custom back sensor with an inertial measurement unit (IMU) from Lifeware Labs LLC (Pittsburgh, PA) was used to capture accelerometer, gyroscope, and magnetometer data (Figure 2A). The sensor, which measures 2.3 in. in length, 1.7 in. in width, and 0.4 in. in thickness, was placed at the level of the L5 spinal segment by a study coordinator (Figure 2). A transparent and waterproof film dressing frame (Tegaderm, 3M, St. Paul, MN) was used to secure the sensor on the skin, ensuring a waterproof and secure placement. Participants were also provided with replacement materials and instructions on how to reattach the sensor if it fell off or needed to be removed temporarily.

Participants also wore two ActiGraph GT9X devices (ActiGraph, Pensacola, FL) (Figure 2B,C), which are tri‐axial accelerometers measuring 1.8 in. by 1.3 in. One device was worn on the nondominant wrist and the other on the waist, attached by a belt. Participants were instructed to wear these devices continuously for 7 days during the daytime, except while showering. They were also instructed to wear their wrist device while sleeping but remove their waist device overnight. After the assessment period, the devices were returned by mail using prelabeled and prestamped envelopes along with the back sensor. The captured data were then synced to cloud‐based CentrePoint software.

### 2.3 Data Processing and Analysis

#### 2.3.1 Self‐Reported EMA Data

The EMA data collected from the app were integrated and stored in a secure remote database. Data were extracted and organized using MySQL and Python. Data were organized based on their time of assessment (morning, afternoon, or evening) and exported into Microsoft Excel format. Sleep duration was calculated from wake‐up time and sleep time information. Descriptive statistics of the data were calculated using Microsoft Excel and Python. The outputs for the EMA data included the distribution and descriptive statistics of pain intensity levels, pain interference levels, activity levels, and sleep duration.

**FIGURE 3:**
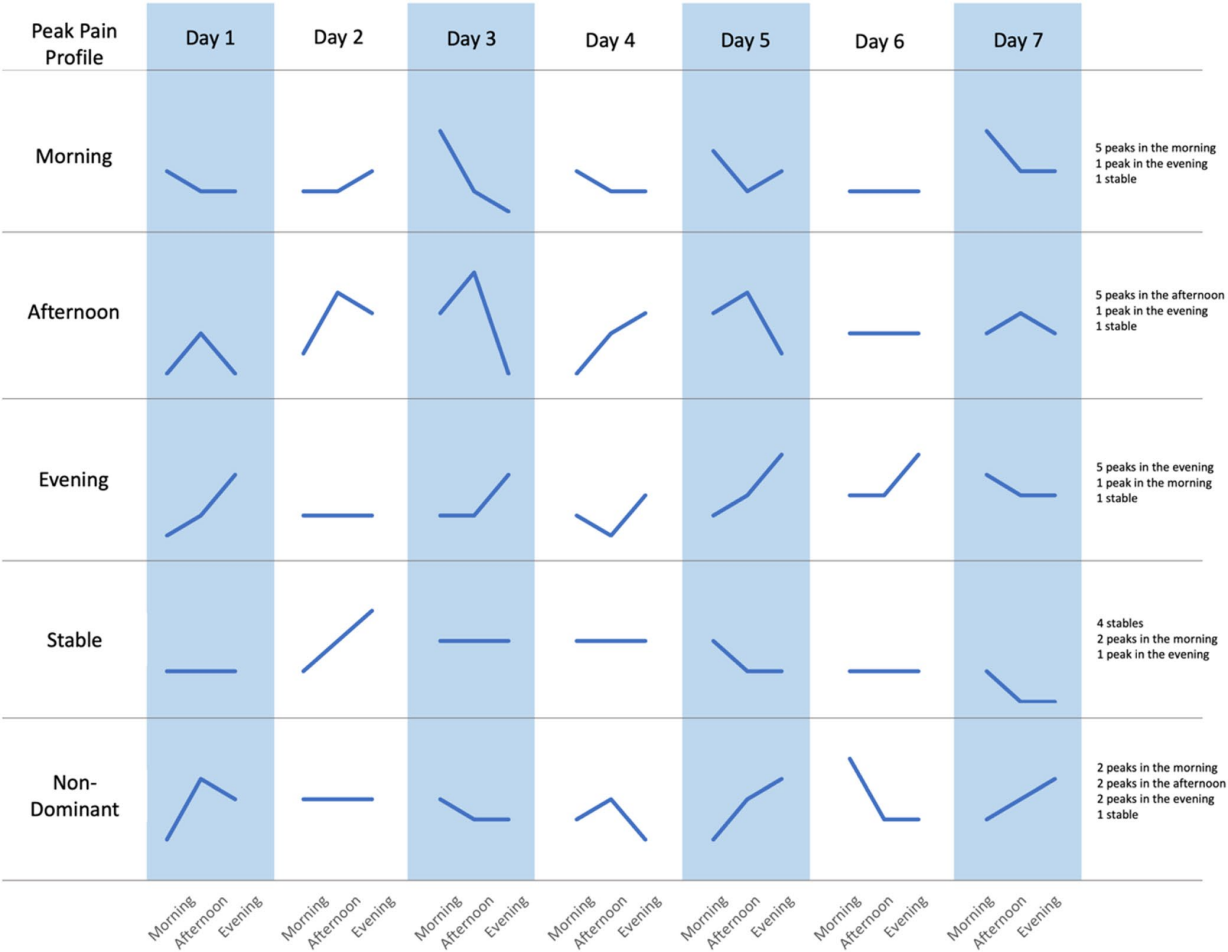
Examples of peak pain categorization based on the daily trend of the pain level during the 7‐day period.

Due to the nature of EMA, granular changes or trends can be observed. Over the 7‐day data collection period, the within‐day changes of pain intensity and pain interference were captured to define when these variables peaked during a given day. Peak pain intensity and pain interference profiles were characterized based on the daily time period during which the majority of daily peak pain profiles were observed during the 7‐day period. Five pain profiles were used: Morning, Afternoon, Evening, Stable, and Non‐Dominant. If there were no changes in pain level during a day, the daily profile for that day was set to Stable. If two or more daily profiles were the majority and occurred with the same frequency during the 7‐day period, making it impossible to determine a single majority, the profile was set to Non‐Dominant. Figure 3 provides an illustration of how the peak pain profile is categorized.

#### 2.3.2 Back Sensor IMU Data

Data from the custom back sensor was collected at 20 Hz and preprocessed using Python. The data was preprocessed by adding timestamp using the information inputted in the app and up sampling the data to 30 Hz to match the ActiGraph algorithm [17]. Activity counts were calculated using ActiGraph's Python package, which were computed in 60‐s epochs [17]. Activity levels were categorized based on Freedson Adult (1998) activity counts cut‐points into sedentary (0–99 counts), light (100–1951 counts), moderate (1952–5724 counts), vigorous (5725–9498 counts), and very vigorous (≥ 9499 counts) levels [18]. Wear time was calculated using the Troiano method [19]. The method defines nonwear time as intervals of at least 60 consecutive minutes of zero activity counts, with allowances for 1–2 min of counts between 0 and 100. Step counts were also calculated using a Python code developed based on the pseudo‐code provided by ActiGraph, which is described and validated by Hoydick et al. [20]. Only data from participants that contained four or more days of data with at least 10 h per day were included in the analysis.

#### 2.3.3 ActiGraph Data

Data from the ActiGraph devices were collected and uploaded to CentrePoint (ActiGraph, Pensacola, FL, USA) using ActiSync (ActiGraph, Pensacola, FL, USA). Those data were then exported from CentrePoint and processed in ActiLife (ActiGraph, Pensacola, FL, USA) to calculate daily metrics for each participant, including wear time, wear‐time‐filtered activity counts in each axis, wear‐time‐filtered step counts, and wear‐time‐filtered activity levels. Filters were used to exclude days with less than 10 h of data, a common threshold for defining days with adequate data [21], and to only include participants with data for at least three weekdays and one weekend day, with a maximum of 7 days included in the analysis [22, 23, 24] Activity counts per day based on vector magnitude, wear time per day, activity levels, and step counts per day were calculated for each sensor (wrist and waist) based on the filtered data. Activity counts per day based on vector magnitude, wear time per day, activity levels, and step counts per day were calculated for each sensor (wrist and waist) based on the filtered data. Activity vector magnitude of the three axes was used to capture movement in multiple directions [19].

### 2.4 Statistical Analysis

Pain intensity level, pain interference level, activity level, and sleep duration were calculated from the self‐reported EMA data. Peak pain intensity profile and peak pain interference profile were also determined. From the back sensor IMU data, activity counts, wear time, activity level, and step counts were calculated. From the ActiGraph devices' data, activity counts in the y axis, wear time, activity level, and step counts were calculated. Sleep duration was also calculated from the wrist ActiGraph. Various metrics exhibited a nonnormal distribution; therefore, medians and interquartile ranges (IQR) were calculated. The medians represent the overall median across all participants, derived from their individual within‐person medians over the 7‐day period. Descriptive statistics for the cohort overall, and stratified by sex (male, female) and age (≥ 60 years old, < 60 years old), are provided.

## 3 Results

### 3.1 Participant Demographics

Out of the 1007 enrolled participants, 989 used the app to submit their EMA data. There were 398 males and 590 females. One participant reported sex at birth as intersex and was therefore not included in the male/female comparison. The age of the participants ranged from 18 to 95 years old with an average of 58.8 ± 16.5 years old, and their BMI averaged 31.5 ± 7.6 kg/m^2^. In terms of racial composition, there were 26 (2.6%) multiracial individuals, 746 (75.4%) White individuals, 18 (1.8%) Asian individuals, 178 (18.0%) Black or African American individuals, 4 (0.4%) individuals classified as Other, and 17 (1.7%) individuals of unknown or undisclosed race. At enrollment, the participants reported a mean pain intensity of 5.4 (SD 2.1) and median pain intensity of 5 (IQR 3) on a 0–10 numeric pain rating scale, and a mean PROMIS Pain Interference *T*‐score of 60.5 (SD 7.5) and median PROMIS Pain Interference *T*‐score of 61.2 (IQR 9.6).

### 3.2 EMA Data

**FIGURE 4:**
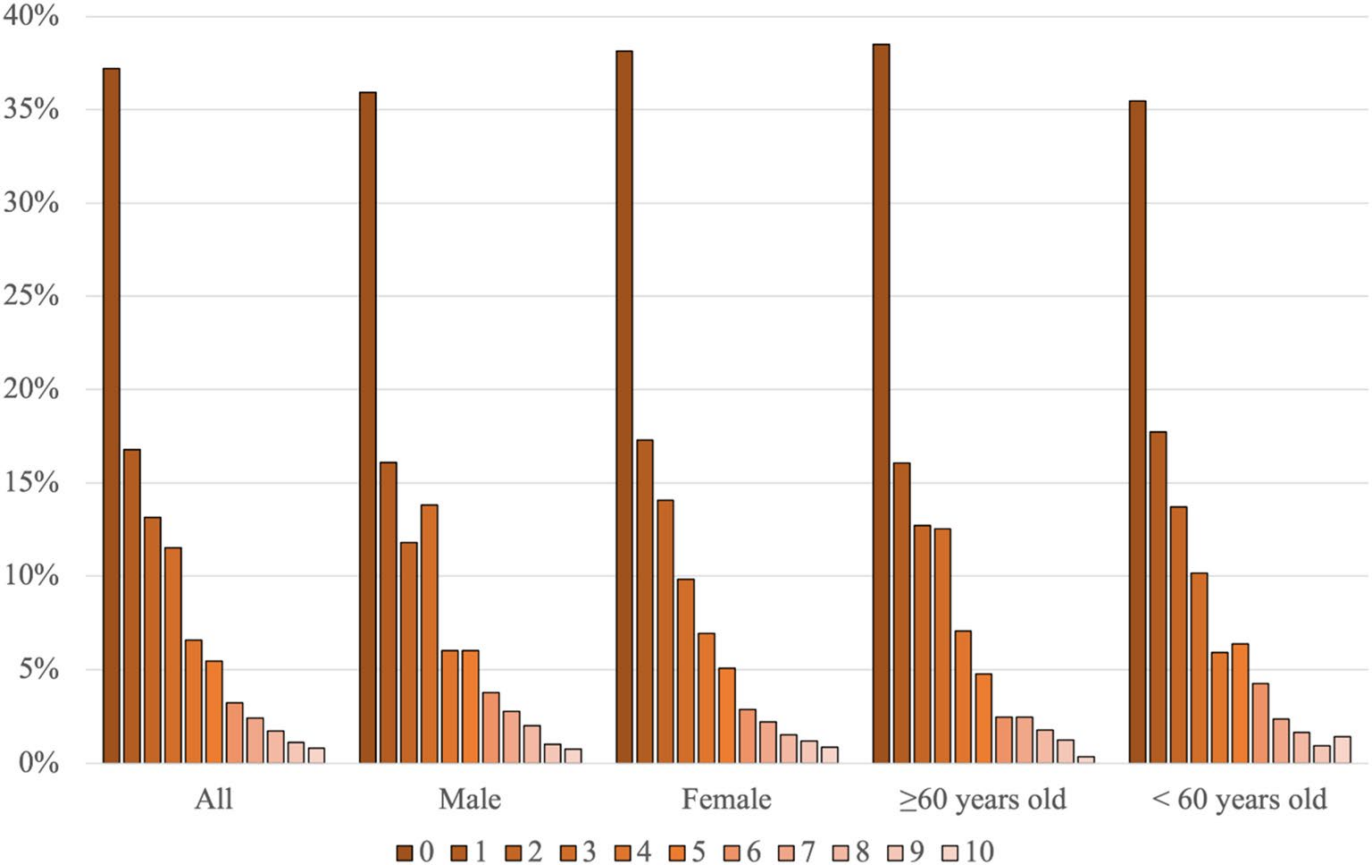
Distribution of median pain intensity level reported in the EMA. Median: overall: 1 (IQR 3); male: 1 (IQR 3); female: 1 (IQR 3); ≥ 60 years old: 1 (IQR 3); < 60 years old: 1 (IQR 3).

**FIGURE 5:**
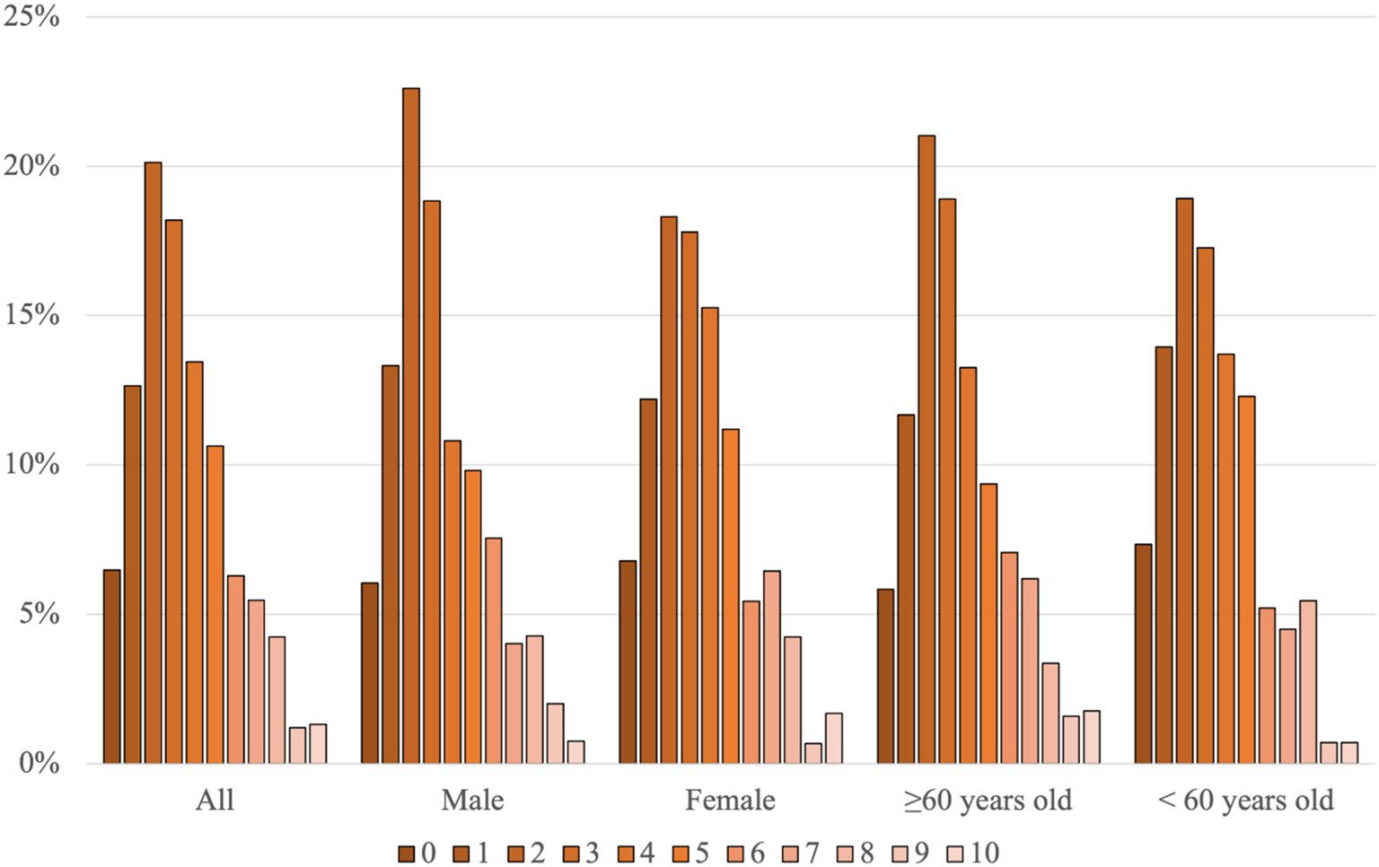
Distribution of median pain interference level reported in the EMA. Median: overall: 3(IQR 3); male: 3(IQR 3); female: 3(IQR 3); ≥ 60 years old: 3(IQR 3); < 60 years old: 3(IQR 3).

More than half of the participants reported a median pain intensity level of either 0 or 1 out of a maximum of 10 (Figure 4). The median of the 7‐day pain intensity levels for the group overall, for males and females, and for those ≥ 60 years old and < 60 years old, was 1 (IQR = 3, range 0–10). The median of the 7‐day pain interference level for the overall group was 3 (IQR = 3, range 0–10). However, a large proportion of participants' median pain interference was 2 out of 10, ranging from 18% of females to 23% of males with this median value (Figure 5).

**FIGURE 6:**
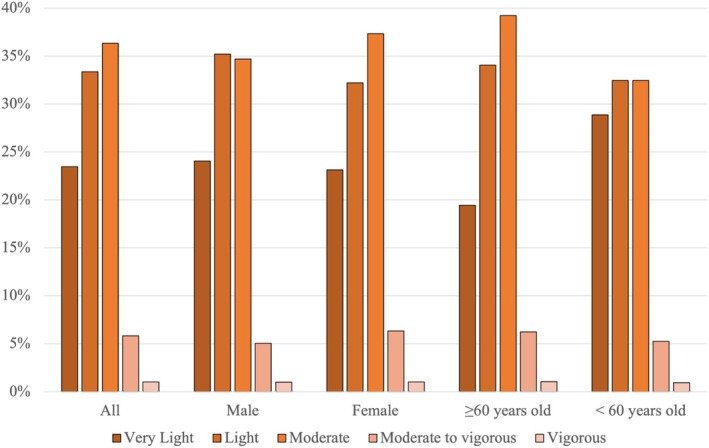
Distribution of median activity level reported in the EMA. Median: overall: light; male: light; female: light; ≥ 60 years old: light; < 60 years old: light.

**FIGURE 7:**
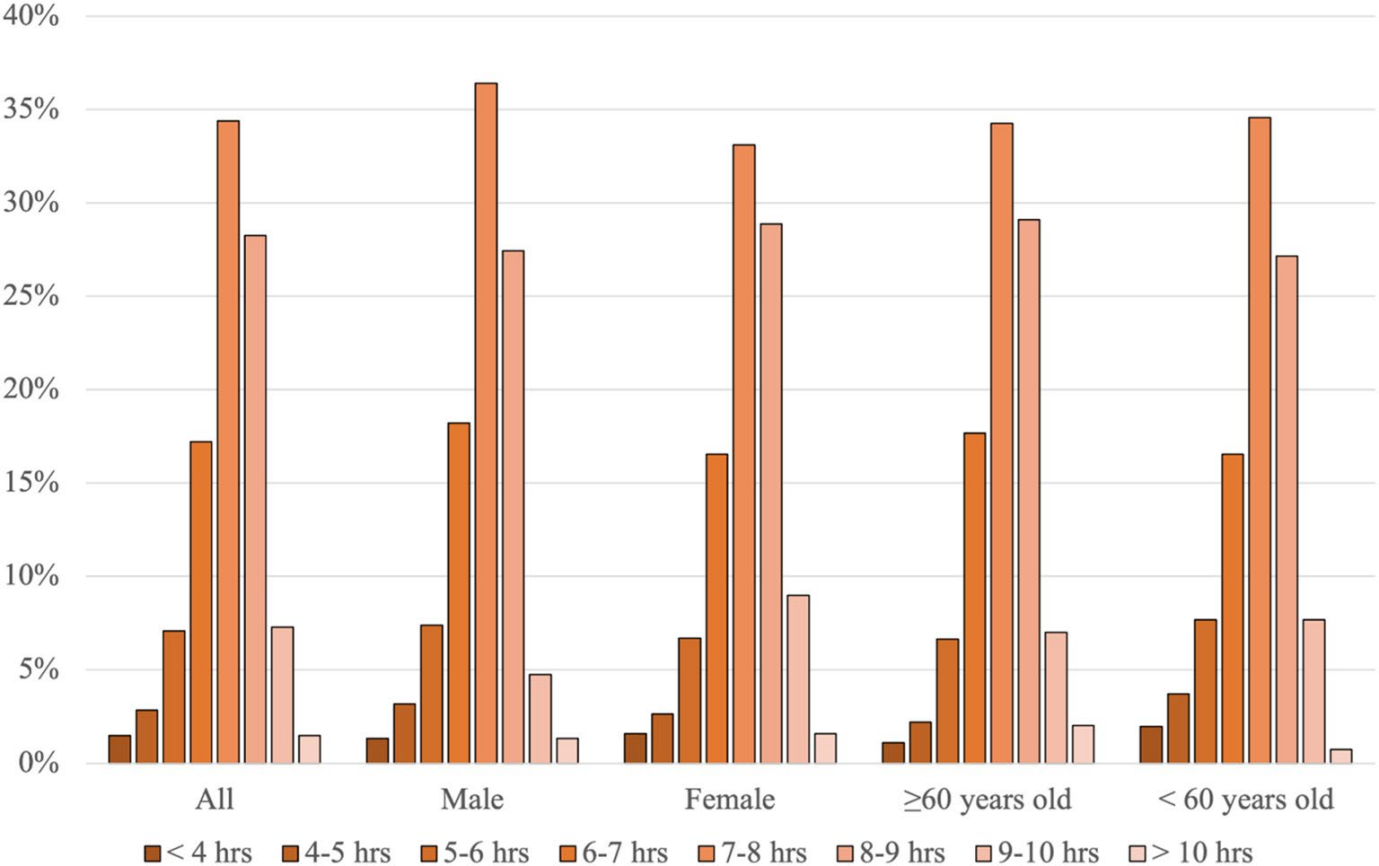
Distribution of median sleep duration self‐reported in the EMA. Mean (hours: minutes ± SD): overall: 7:29 ± 1:14; male: 7:24 ± 1:12; female: 7:32 ± 1:16; ≥ 60 years: 7:32 ± 1:12; < 60 years: 7:24 ± 1:18.

For activity level, a third of participants (36%) reported a 7‐day median of moderate activity level, while 33% of participants reported a 7‐day median of light activity level (Figure 6). Findings were similar for males and females. However, in the age groups, the < 60 years old group had a median of moderate to vigorous activity level (39%). Approximately one‐third of the participants (34%) reported a median sleep duration between 7 and 8 h (Figure 7).

**FIGURE 8:**
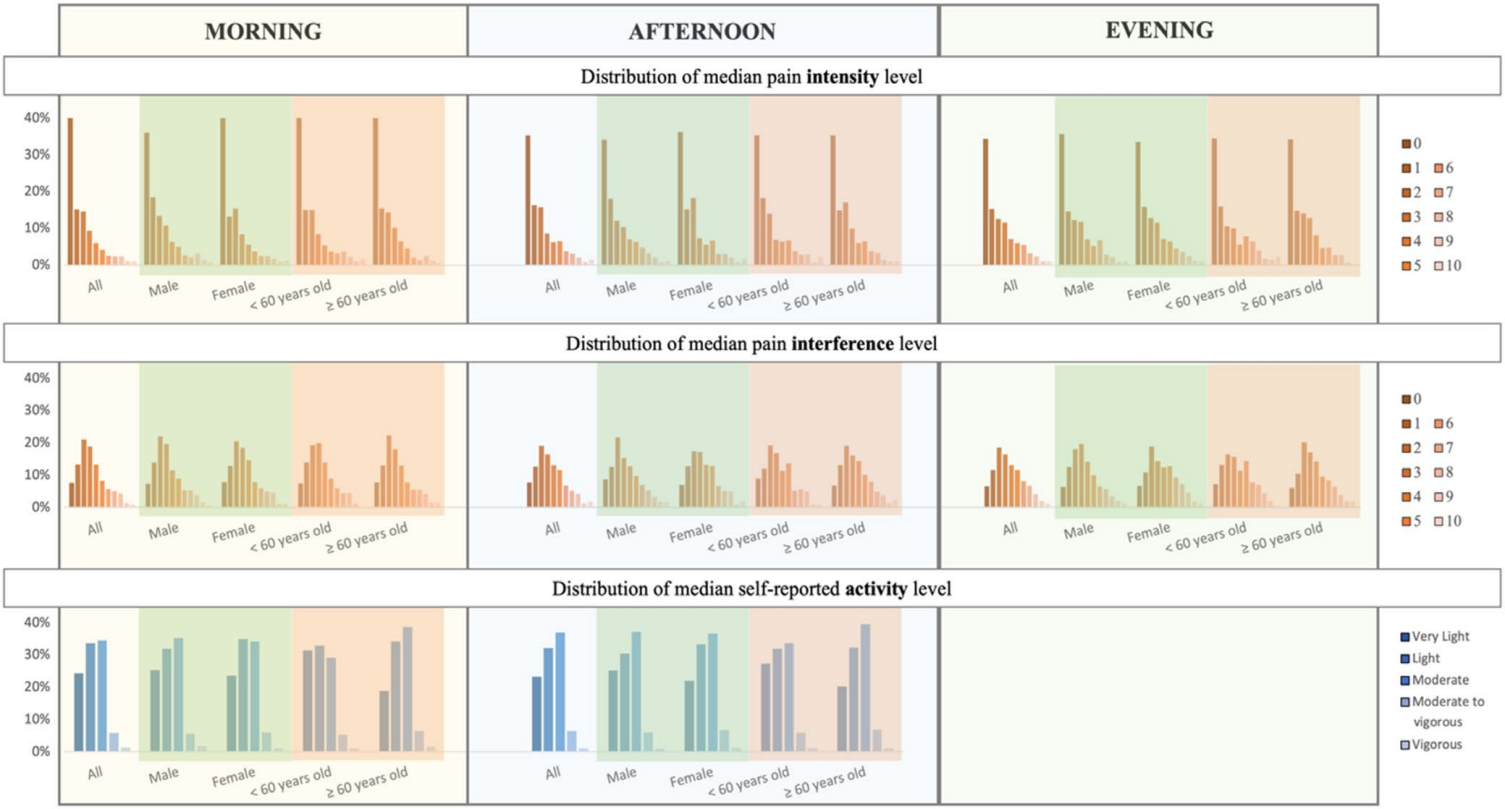
Distribution of median pain intensity level, median pain interference level, and median activity level in the morning (left), afternoon (middle), and evening (right) reported in the EMA over the 7‐day at‐home assessment period. Median pain intensity was 1 (IQR 3) across all time points, except for males and participants under 60 years old in the afternoon and evening (1 [IQR 4]); overall and female participants in the evening (2 [IQR 4]); and participants aged 60 years and older in the evening (2 [IQR 3]). Median pain interference was 3 (IQR 3) across all categories. Median activity level was reported as “light” for all categories.

For pain intensity in the morning, 90.7% of participants reported a median pain intensity level between 0 and 5 (mild), 4.8% reported a median pain intensity level between 6 and 7 (moderate), and 4.4% reported a median pain intensity between 8 and 10 (severe). In the afternoon, 88.7% of participants reported a median pain intensity level between 0 and 5, 6.9% reported a median pain intensity level between 6 and 7, and 4.4% reported a median pain intensity between 8 and 10. In the evening, 86.8% of participants reported a median pain intensity level between 0 and 5, 8.7% reported a median pain intensity level between 6 and 8, and 4.4% reported a median pain intensity between 9 and 10 (Figure 8).

For pain interference levels in the morning, 82.6% of participants reported a median pain interference level between 0 and 5, 10.8% reported a median pain interference level between 6 and 7, and 6.6% reported a median pain interference between 8 and 10. In the afternoon, 80.7% of participants reported a median pain interference level between 0 and 5, 12.0% reported a median pain interference level between 6 and 7, and 7.3% reported a median pain interference between 8 and 10. In the evening, 77.9% of participants reported a median pain interference level between 0 and 5, 14.8% reported a median pain interference level between 6 and 8, and 7.3% reported a median pain interference between 8 and 10 (Figure 8).

For activity level, in the afternoon, most participants across all groups reported a moderate activity level. Variability was observed in the morning activity levels. Most participants in the female group and the < 60 years old group reported a light activity level (Figure 8).

Regarding trajectories of pain intensity and interference across days, most participants across all groups experienced peak pain intensity in the evening. The second‐largest proportion of participants in all groups had a stable pain intensity profile (Table 2). For pain interference, similar to pain intensity, the largest proportion of participants in all groups reported peak levels in the evening. However, the second‐largest proportion in almost all groups reported peak pain interference in the morning, except for the < 60 years old group, for which nondominant pain interference was the second most frequently occurring profile (Table 3).

**TABLE 2:** Distribution of pain intensity level profile based on time when pain peaks during the day.

Peak pain intensity profile^a^	All (*N* = 901)	Male (*N* = 362)	Female (*N* = 538)	≥ 60 years old (*N* = 524)	< 60 years old (*N* = 377)
Stable	24.1%	25.7%	23.0%	24.6%	23.3%
Morning	14.4%	18.2%	11.9%	14.7%	14.1%
Afternoon	17.0%	15.5%	18.0%	19.1%	14.1%
Evening	30.2%	27.1%	32.3%	30.3%	30.0%
Non‐dominant	14.3%	13.5%	14.7%	11.3%	18.6%

^a^ Peak pain intensity profiles were categorized based on the majority of daily peak pain intensity profiles over 7 days. The five profiles used were Morning (peak in the morning), Afternoon (peak in the afternoon), Evening (peak in the evening), Stable (no changes in pain intensity level during a day), and Non‐Dominant (no single majority profile over 7 days).

**TABLE 3:** Distribution of pain interference level profile based on time when pain interferes most during the day.

Peak pain interference profile^a^	All (*N* = 901)	Male (*N* = 363)	Female (*N* = 538)	≥ 60 years old (*N* = 524)	< 60 years old (*N* = 377)
Stable	8.9%	11.9%	6.9%	9.0%	8.8%
Morning	20.5%	24.9%	17.7%	21.8%	18.8%
Afternoon	14.2%	12.4%	15.4%	15.5%	12.5%
Evening	40.5%	36.7%	43.1%	41.6%	39.0%
Non‐dominant	15.9%	14.1%	16.9%	12.2%	21.0%

^a^ Peak pain interference profiles were categorized based on the majority of daily peak pain interference profiles over 7 days. The five profiles used were Morning (peak in the morning), Afternoon (peak in the afternoon), Evening (peak in the evening), Stable (no changes in pain interference level during a day), and Non‐Dominant (no single majority profile over 7 days).

### 3.3 Physical Activity From Back Sensor and ActiGraph Devices

**FIGURE 9:**
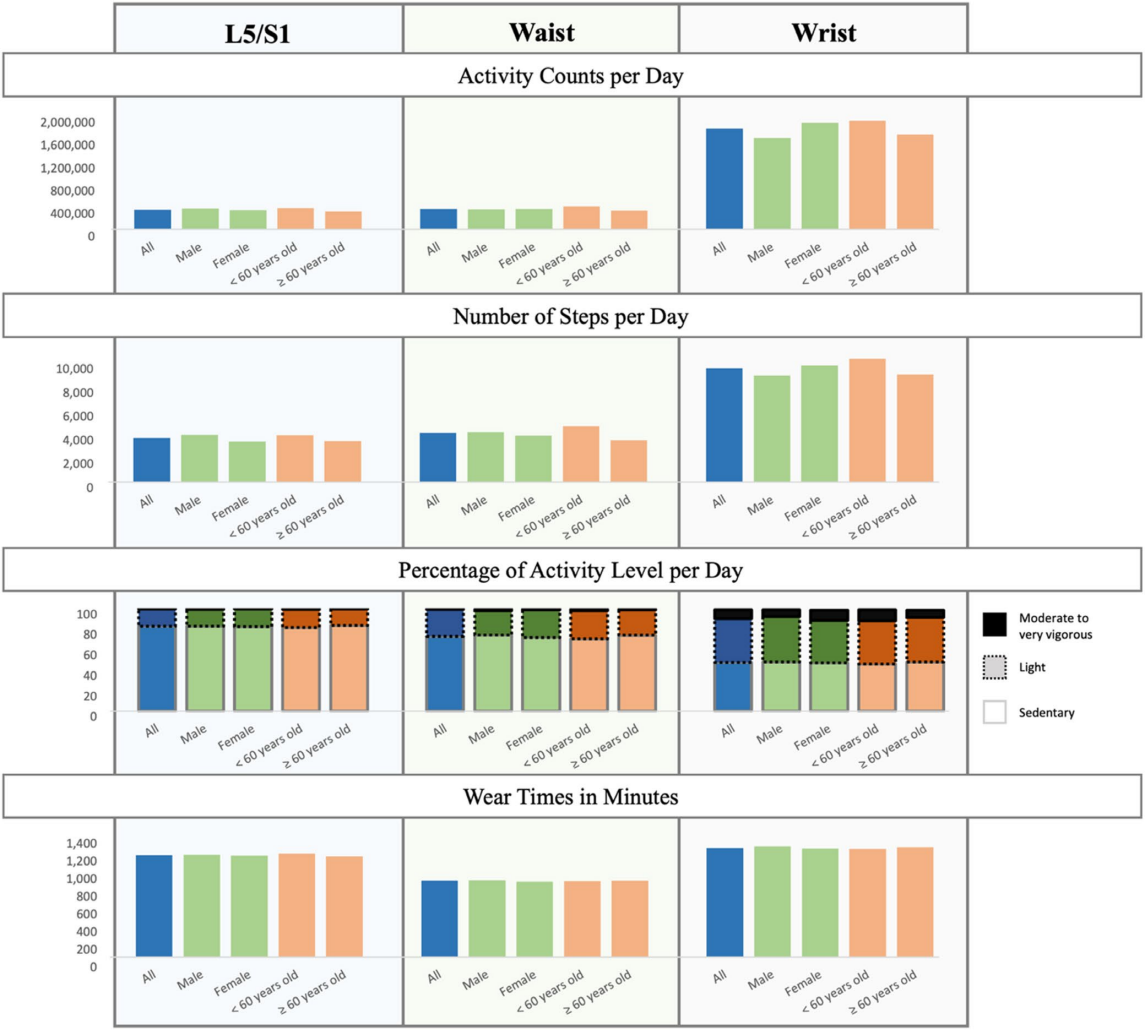
Physical activity calculated from L5 back sensor, wrist ActiGraph, and waist ActiGraph for participants with minimum 4 days of data and 10 h of wear time per day. For L5 back sensor, *N*: all = 586, male = 244, female = 341, < 60 years old = 230, and ≥ 60 years old = 356. For wrist ActiGraph, *N*: all = 884, male = 364, female = 519, < 60 years old = 350, and ≥ 60 years old = 534. For waist ActiGraph, *N*: all = 785, male = 317, female = 467, < 60 years old = 295, and ≥ 60 years old = 490. Wear time refers to the period during which the sensors were actively recording data, excluding periods of inactivity due to lack of movement from the participants, such as during sleep.

For the back sensor analysis, after filtering the data to include only participants with at least 3 weekdays and 1 weekend day with more than 10 h in each day, 586 participants (244 males and 341 females; 356 ≥ 60 years old and 230 < 60 years old) were included in the analysis. The median activity counts per day were 340 345 (IQR 223 399) (Figure 9). The median step counts were 3695 (IQR 2743) (Figure 9). Qualitatively, the < 60 years old group exhibited more activity counts and step counts compared to the ≥ 60 years old group.

More than 80% of participants across all groups had a median activity level classified as sedentary (Figure 9). A median activity level of light activity was observed in 17% of participants. Similar to activity counts and step counts, qualitatively, the male and < 60 years old groups spent more time in moderate to very vigorous activity levels relative to females and ≥ 60 years old groups, respectively.

Similar to the back sensor analysis, in the ActiGraph data analysis, the number of participants was reduced due to the requirement to have a minimum of 4 days of data with at least 10 h of wear time each day. For ActiGraph worn on the wrist, 884 participants (364 male and 519 female; 534 ≥ 60 years old and 350 < 60 years old) were included, while for ActiGraph worn on the waist, 785 participants (317 male and 467 female; 490 ≥ 60 years old and 295 < 60 years old) were included.

The median activity counts per day calculated from the wrist ActiGraph data were 1 765 325 (IQR 796 995) (Figure 9). For waist ActiGraph data, the median activity counts were 358 390 (223 758) (Figure 9). The median step counts were 9575 (IQR 4228) for the wrist and 4114 (IQR 3146) for the waist. The group of < 60 years old qualitatively had more step counts relative to the ≥ 60 years old participant group. The female group generally had higher activity counts and step counts relative to the male group based on the wrist data.

The largest percentage of waking time was spent in the sedentary activity level. The waist sensor data demonstrated that around 70% of the activity time was sedentary, while the wrist sensor data demonstrated comparable percentages between the sedentary and light activity levels, at 47.3% and 42.7%, respectively. Based on the waist data, generally, males and the < 60 years old group spent more time in moderate to very vigorous activity levels compared to females and the ≥ 60 years old group, respectively. However, the wrist data showed a higher percentage of time spent in moderate to very vigorous activity level in the female group compared to the male group.

## 4 Discussion

Individuals with cLBP often experience significant disability and reduced activity levels. Understanding the daily experiences of individuals with cLBP is crucial for characterizing the impact of cLBP and developing effective interventions. Therefore, the objective of this study was to characterize pain characteristics, physical activity, sedentary behavior, and sleep patterns from individuals with cLBP in the field over a 7‐day period.

The self‐reported EMA results showed that the participants frequently reported low pain intensity and interference levels, which was unexpected for a cohort of individuals with cLBP and differed from their self‐reported pain score at the time of enrollment in the study. More than half of the participants reported a 7‐day median pain intensity of either 0 or 1 (54.0%) and pain interference between 0 and 3 (57.4%). It should be noted that participants were asked to report their pain intensity and pain interference levels as they were experienced “right now,” which indicates that, at some point during the collection period, the majority of participants had periods of little to no pain. This result also differed from external research that collected pain intensity from a low back pain population in their one‐a‐day EMA, where the average pain intensity was 3.5 (noting this average pain value cannot be directly compared to the median value reported in the present study) [25].

In contrast, when the same cohort of 989 individuals with cLBP were asked to self‐report their pain intensity and pain interference levels over the previous week using patient‐reported outcome measures at their enrollment study visit, they reported a mean pain intensity of 5.4 (SD 2.1) and median pain intensity of 5 (IQR 3) on a 0–10 numeric pain rating scale, and a mean PROMIS Pain Interference *T*‐score of 60.5 (SD 7.5) and median PROMIS Pain Interference *T*‐score of 61.2 (IQR 9.6), which is one standard deviation from the population norm of 50 and represents moderate impairment [26]. This discrepancy may result from the difference in time frame of the questions (right now vs. past 7 days), reflects the variability in pain throughout the day, and points to the need to reexamine the timeframe and mechanism by which these outcomes are assessed. These findings also underscore the importance of collecting self‐reported EMA data which may more accurately capture the variability in a person's pain experience and set the stage for ecological momentary interventions.

When the self‐reported EMA pain and pain interference were summarized to determine the pain and pain interference peak time during the day, the most frequent peak pain profile was the evening for both pain intensity (27%–30%) and pain interference (37%–43%) across both sex and age groups. A profile of peak pain in the evening suggests that, for many participants, pain accumulates or amplifies over the day. Previous studies investigating the circadian rhythms in chronic pain patients suggest that a more thorough understanding of the pain rhythms can facilitate the performance of daily activities and physical exercise based on the time of the day, leading to better pain management [27].

In terms of activity levels assessed via self‐reported EMA, individuals with cLBP in this cohort most frequently reported a light activity level. However, the younger (< 60‐year‐old) group most frequently self‐reported a moderate to vigorous activity level. These self‐reported results are in sharp contrast to the activity levels determined from the sensor data wherein individuals with cLBP were sedentary for 49%–83% of their time. These findings are consistent with the literature that shows that both individuals with cLBP and non‐cLBP individuals tend to underestimate their sedentary time and overestimate their time spent in vigorous activities [27]. Individuals with cLBP have been shown to underestimate their sedentary time even more than those without cLBP [27]. Moreover, the correlation between self‐reported activity levels and objective activity levels in individuals with cLBP is weak [28]. The broad range of time spent in sedentary activities (47%–83%) was impacted significantly by the sensor wear locations, with the wrist showing the lowest percentage (47%) and the lumbar region showing the highest percentage (83%). This may point to the need to re‐evaluate the activity level cut‐points according to wear location. Further research is needed to better understand the impact of these differences for assessment of activity levels for individuals with cLBP. Regardless of the discrepancy between the self‐reported and sensor‐assessed activity levels and the wide variability due to wear location, it is important to encourage sedentary cLBP individuals to increase their activity levels. Their activity counts as calculated by the waist ActiGraph and the back sensor were lower than the average activity counts in the US population older than 16 years old, which is around 489 357 counts per day [21].

Wear location also resulted in differences in the magnitude of activity counts. On average, the number of activity counts recorded for the wrist‐worn location was approximately 4–5 times greater than that observed at the waist‐worn location. These results are consistent with previous literature showing that wrist‐worn devices are more susceptible to noise from arm and hand movements, leading to an overestimation of activity counts and corresponding activity levels [13]. The lower back wear location resulted in activity count numbers that were comparable to the waist‐worn locations. Future work is needed to determine how these activity counts are associated with each other and clinical outcomes for individuals with cLBP.

Similar differences were observed in step counts across wear locations. Step counts recorded from the wrist‐worn device were more than twice those recorded from both the waist‐worn and lower back‐worn devices. The number of step counts recorded at the waist and lower back locations was similar in magnitude. Little work has been done to evaluate the psychometric properties of accelerometers for measuring step counts in people with cLBP; however, research studies have explored the benefits of walking as a management strategy for cLBP [29].

In terms of sex and age differences, the younger age group (< 60 years old) tended to display higher activity counts and step counts overall regardless of wear location. This is consistent with previous studies showing that these factors are inversely correlated with age [30]. Little to no differences were observed for activity counts and step counts between sexes. Although the data were presented by sex and age categories, the results and discussion sections only provided qualitative comparisons. Future work is planned to conduct inferential analyses for this cohort.

Sleep duration was estimated from the self‐reported EMA data as the difference between the time the participants reported they fell asleep and the time they woke up. Based on the self‐reported EMA data, the most frequent median sleep duration was 7–8 h, which is slightly higher than the average sleep duration of 6.5 h per night participants reported at the study enrollment visit [26]. Although both of these data points fall within the normal ratings for sleep duration, approximately 10%–20% of participants reported less than 6 h of sleep per night, which is consistent with approximately 20% of the overall sample that reported moderate to severe sleep disturbance at baseline on the PROMIS survey [26]. Previous studies have found that multiple dimensions of sleep are adversely associated with cLBP, indicating that sleep parameters may be important for phenotyping and management of individuals with cLBP [31].

One limitation of the activity monitoring using the custom back sensors in this study was that data could be collected only from approximately 60% of participants to meet the minimum of 4 days and 10 h of wear time per day. There were numerous factors that contributed to this data loss, including both sensor‐related factors (i.e., sensor availability and sensor malfunctions) and participant‐related factors (i.e., skin irritation and acceptability). This is consistent with the findings of a narrative review that indicated that accelerometers placed on the wrist, waist, thigh, ankle, and foot are more acceptable to users than those placed on the neck, chest, trunk, elbow, and fingers [32]. Future work is required to improve the wearability and acceptability of sensors worn on the lower back and/or to determine if the benefits of this wear location are sufficient to justify the increased participant burden.

This study provides valuable insights into the daily physical activity, sedentary behavior, and sleep patterns of individuals with cLBP through the use of wearable sensors and self‐reported EMA. By leveraging data from a large, diverse cohort, this work underscores the feasibility and importance of integrating real‐world biomechanical, behavioral, and self‐reported data to characterize the multifaceted impact of cLBP on daily life. Ongoing efforts aim to integrate these findings with additional biological, behavioral, and biomechanical factors to identify distinct cLBP phenotypes. Furthermore, the development of predictive models using these datasets could facilitate the design of tailored therapeutic approaches aimed at improving outcomes for individuals with cLBP.

## Funding

This work was supported by the National Institute of Arthritis and Musculoskeletal and Skin Diseases (U19AR076725‐01).

## Conflicts of Interest

The authors declare no conflicts of interest.
